# Effects of Repeated Monopolar Pulsed Capacitive Radiofrequency Energy Treatments on Mechanical Nociceptive Thresholds and Skin Temperature in Show-Jumping Horses with Thoracolumbar Pain

**DOI:** 10.3390/ani16182943

**Published:** 2026-09-19

**Authors:** Benedetta Brenna, Maria Chiara Pressanto, Marco Pepe, Bruno Boccioli, Stefanie Rouge, Francesca Beccati

**Affiliations:** 1 Department of Veterinary Medicine, University of Perugia, Via san Costanzo 4, 06126 Perugia, PG, Italy; benedetta.brenna@gmail.com (B.B.); chiarapressanto.dvm@gmail.com (M.C.P.); marco.pepe@unipg.it (M.P.); 2 Sports Horse Research Centre, University of Perugia, Via san Costanzo 4, 06126 Perugia, PG, Italy; 3 Radio Frequency Consulting, Via Dante Alighieri 44, 06012 Città di Castello, PG, Italy; bruno.boccioli@gmail.com; 4 Private Practice, Via delle Maestà 29, 06063 Magione, PG, Italy; stefanie.rouge@hotmail.it

**Keywords:** back pain, capacitive and resistive energy transfer, pressure algometry, radiofrequency, show jumping horses, show-jumping, showjumping, show jumping, thermography

## Abstract

This study evaluated the effects of repeated monopolar Capacitive Resistive Electrical Transfer (CRET) therapy on mechanical nociceptive thresholds (MNT) and skin surface temperature in horses with perceived chronic thoracolumbar pain. Fifteen show-jumping horses were enrolled in a randomized sham-controlled clinical trial. Ten horses received treatment, and five underwent a sham procedure. Five treatment sessions were performed at 72 h intervals using a monopolar capacitive radiofrequency device operating at 500 kHz. Mechanical nociceptive thresholds (MNT) were assessed before treatment, after each session, and during a 45-day follow-up period. Infrared thermography was performed before and after each treatment session. MNT data were analyzed using linear mixed-effects models including treatment group, time, anatomical site and their interactions as fixed effects. CRET treatment resulted in a progressive increase in MNT, with significant effects of treatment group, time and their interaction (*p* < 0.0001). Higher ΔMNT values were observed in treated horses from the second treatment session onwards and remained above baseline throughout the follow-up period. Skin surface temperature increased after both CRET and sham procedures, with no significant difference between groups. Repeated monopolar capacitive CRET increased MNT in show-jumping horses with perceived chronic thoracolumbar pain, while producing no greater immediate thermographic response.

## Simple Summary

Back pain is common in sport horses and may negatively affect athletic performance and welfare. Currently, there is growing interest in non-invasive physical therapies that may support the management and rehabilitation of horses with back problems while limiting the need for pharmacological treatments. This study investigated the effects of radiofrequency-based treatment in show-jumping horses with a history of long-term back pain in 15 horses. Ten received five treatment sessions at three-day intervals, while five underwent an identical procedure without activating the device. Back sensitivity was evaluated by measuring the amount of pressure required to produce a response at several locations along the back. Measurements were performed before treatment, after each session, and every 15 days for 3 additional evaluations after completion of the treatment period. Skin temperature was also evaluated before and after each procedure using thermal imaging. Horses receiving radiofrequency treatment showed a progressive increase in the amount of pressure required to elicit a response, which remained above initial values throughout the 45-day follow-up period. In contrast, little change occurred in untreated horses. Changes in skin temperature were similar between groups. These findings warrant further investigation of radiofrequency therapy as a non-invasive rehabilitation option for sport horses with back problems.

## 1. Introduction

In equestrian sports, the feature of show jumping horses lies in their ability to tackle obstacles, requiring intense and repetitive physical exertion. However, these physical demands often result in the clinical development of back pain, as the biomechanics of jumping places substantial stress on the equine back, increasing the risk of chronic pain and associated musculoskeletal disorders [1]. Due to repetitive mechanical loading of the back in show jumpers, back pain may be muscular in origin or also be related to issues involving the facets joints (i.e., osteoarthritis) or the dorsal spinous processes (i.e., kissing spine) or the sacroiliac joint dysfunction [2]. These issues can limit athletic performance and also adversely impact the horse’s welfare [2].

Traditional treatment protocols for equine back pain generally include the identification of any underlying pathology, the administration of pain-relieving drugs, typically non-steroidal anti-inflammatory drugs, mesotherapy, intra-muscular/ligamentous injections, ultrasound-guided articular process joint injections, structured rehabilitation program, evaluation of saddle fit and horse–rider interaction, along with physical therapies—such as massage, electrical stimulation, shockwave therapy, and water treadmill exercise—to promote healing and expedite recovery [2,3,4].

Radiofrequency energy encompasses therapeutic modalities based on the application of high-frequency electromagnetic energy to biological tissue. Depending on the operating frequency, diathermy systems are generally classified as long-wave (3–300 kHz), shortwave (3–30 MHz), or microwave (300–3000 GHz) devices [5]. Within the radiofrequency spectrum, however, different therapeutic technologies coexist, and terminology is not always used consistently across the literature [6,7,8]. These approaches may differ substantially in terms of invasiveness, carrier frequency, continuous or pulsed/modulated emission, electrode configuration, and the resulting thermal and non-thermal biological effects [6,7,8].

In human literature, Capacitive Resistive Electrical Transfer (CRET) is a non-invasive endogenous electrothermic therapy based on the application of high-frequency alternating radiofrequency currents through capacitive and/or resistive energy transfer. CRET is also described in the human literature as capacitive–resistive monopolar radiofrequency (CRMRF), while the term TECAR is frequently used in clinical and commercial settings to describe related capacitive–resistive energy-transfer technologies. Commercial systems generally operate within the range of approximately 300–1200 kHz, depending on the manufacturer and clinical application [9,10,11]. Importantly, CRET systems may deliver radiofrequency energy continuously or using modulated/pulsed emission, resulting in different contributions of thermal and non-thermal bioelectrical effects [10,12]. The technology delivers radiofrequency energy through active and return electrodes, generating endogenous heat within biological tissues according to their electrical impedance, while also inducing non-thermal bioelectrical effects [9,10,11,12,13,14,15]. The biological effects of CRET are generally attributed to two complementary mechanisms. The first consists of bioelectrical effects produced by the alternating current itself, which have been associated with modulation of cell signaling pathways, proliferation of mesenchymal stem cells and regulation of inflammatory processes, even under conditions of minimal heat generation [12,14,16]. The second mechanism is related to endogenous tissue heating resulting from energy dissipation within biological tissues due to electrical resistance of the tissues, leading to increased local blood flow, tissue oxygenation, metabolic activity, collagen extensibility and pain modulation [12,14,16]. CRET devices typically include capacitive and resistive modes of energy transfer; capacitive application preferentially concentrates energy in soft tissues with higher water content, such as muscles, cartilage, and adipose tissue, whereas resistive application delivers greater energy to tissues with higher electrical resistance, such as tendons, ligaments, fascia and bone interfaces [9,10,12,16].

In equine rehabilitation, CRET has attracted increasing interest over the last few years [13,17,18,19,20]. Experimental studies demonstrated increases in superficial temperature and prolonged thermal responses depending on treatment intensity, supporting its use as a deep thermotherapy modality [19,20]. Additional investigations have reported improvements in stride characteristics and accelerometric variables both during treadmill exercise and in athletic horses performing discipline-specific exercise, suggesting potential effects on locomotor biomechanics and performance [13,17,21]. Clinical studies have further described reductions in thoracolumbar pain and improvements in back flexibility following repeated CRET sessions [18,20].

Although CRET has been increasingly investigated in equine rehabilitation, current evidence mainly focuses on thermographic responses, locomotor biomechanics and subjective evaluations of pain and flexibility [13,17,18,19,20,21]. To the authors’ knowledge, no randomized case–control study has evaluated the effect of a capacitive monopolar radiofrequency protocol on thoracolumbar pain in show-jumping horses using objective mechanical nociceptive threshold measurements. We hypothesized that (i) the treatment of the epaxial muscles would increase the mechanical nociceptive threshold (MNT) in the back, and (ii) the procedure would result in an increase in skin temperature, as measured by thermographic imaging, in both cases and controls.

## 2. Materials and Methods

### 2.1. Horses

This study was approved by the Bioethical Committee of the University of Perugia with the number 196312. Horses were excluded if they had severe systemic diseases, acute or chronic injuries affecting the appendicular skeleton (i.e., forelimb or hindlimb lameness), or if they had received treatments for back pain within the six weeks prior to the study. Fifteen horses used as show jumpers with a perceived history of back pain of at least 6 months in the thoracolumbar region and specifically in the saddle area, reported by their owners, riders or referring veterinarians were included in the study. All horses underwent a thorough clinical and lameness examination to confirm a functional diagnosis of back pain based on the assessment of back pain, stiffness, and increased muscle tone by manual palpation; they were also assessed as clinically healthy by a board certified in sports medicine and rehabilitation (FB); no diagnostic imaging examinations were performed. “Clinically healthy” was defined as the absence of abnormalities in vital parameters, including respiratory and heart rate, peripheral pulse, skin elasticity, mucous membrane color, temperature and enlargement of the mandibular and retropharyngeal lymph nodes. The muscle condition score (MCS) of each horse was evaluated using a previously published grading system [22].

Randomly, 10 horses were allocated in the treatment group (group t) and five horses in the control group that underwent a sham treatment (group s).

Horses did not follow a standardized rehabilitative exercise protocol. On the days of CRET therapy session or sham procedure, they were either rested. Throughout the study period, horses were limited to flatwork only, with no participation in competitions or jumping training sessions. No additional treatments were performed during the study’s execution.

### 2.2. CRET Treatments Protocol

CRET treatment was delivered using a monopolar handheld device (Top Quality Vet, Top Quality group S.r.l., via G. Sorel, Città di Castello, PG, Italy). This system features dual electrical insulation, a maximum power output of 150 Watts, and an impedance of 50 ohms. The operating frequency can be adjusted via software control, with a range between 500 kHz and 1500 kHz. In this study, the device was operated at 500 KHz. The device can deliver energy through capacitive and resistive modes from the same handheld; however, only the monopolar capacitive mode was used throughout the study. The active electrode measured 3 cm in diameter, while receiving plate measured 10 × 5 cm. A monopolar configuration was selected because it generates broader and deeper electromagnetic fields than bipolar systems, making it more suitable for reaching thoracolumbar epaxial musculature [9].

**Figure 1:**
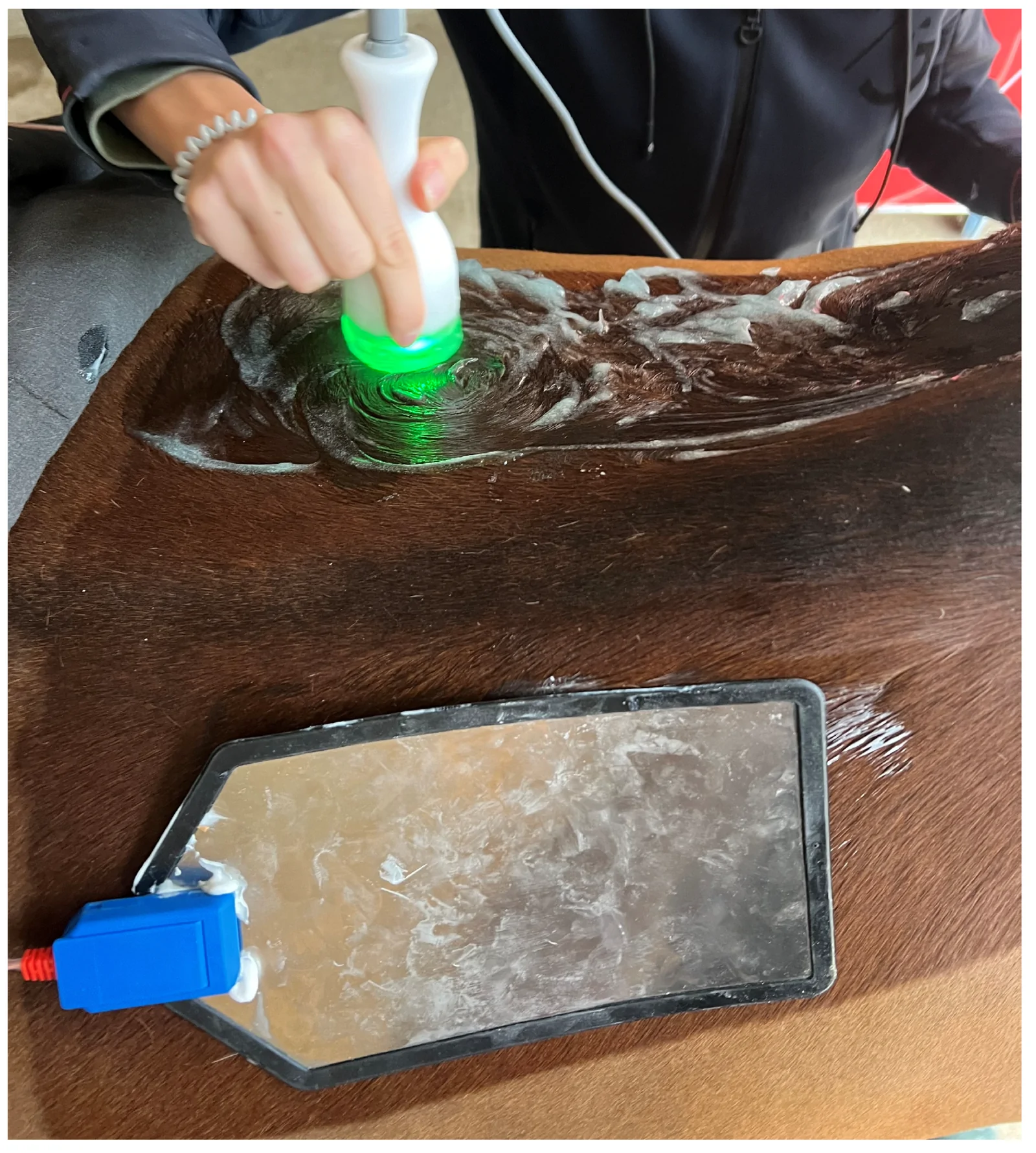
Monopolar capacitive CRET treatment applied to the saddle region, targeting the thoracolumbar epaxial musculature. The active handheld electrode was maintained in direct contact with the treatment area and moved using slow circular movements. Note the return electrode positioned on the contralateral side of the thoracolumbar region.

None of the horses were sedated either for the treatment or sham procedures. Prior to each session, the dorsal region (i.e., back) of the horses was cleaned with brush, wet with tap water applied using a sponge and a uniform layer of conductive gel (ambient temperature) was applied to both the treatment area and the receiving plate to reduce cutaneous impedance and optimize electrode-skin contact, minimizing coupling interference [9,23]. Care was taken to avoid excessive gel application, which could alter current dispersion and affect electrode stability [23]. The receiving plate was positioned contralaterally to the treatment area and reposited symmetrically when switching sides (Figure 1).

Treatment sessions were performed every 72 h for a total of five applications (T1–T5). The treatment was applied to the saddle region, specifically the epaxial area between the ninth thoracic (Th9) and seventeenth thoracic (Th17) vertebrae, with these anatomical locations estimated by palpation and counting the ribs, targeting the *longissimus dorsi*, spinalis dorsi, and iliocostal muscles. All treatments were performed by the same veterinarian to minimize operator-dependent variability. The operator maintained consistent manual pressure, electrode orientation and movement speed thought the session, as these factors are known to influence thermal patterns during radiofrequency application [9]. The handheld device was moved in slow, circular motions along the thoracolumbar region in both cranio-caudal and caudo-cranial directions, ensuring uniform coverage of the area of interest. Movement speed was kept as constant as possible across sessions to improve comparability. Each session lasted 20 min, with equal time allocated to the right and left sides of the back.

The treatment intensity was adjusted according to the muscle condition score (MCS) of each horse, with a feedback power of 35% for MCS of 4, 33% for MCS of 3 and 30% for MCS of 2. This adjustment of the treatment protocol was based on the authors’ clinical experience and manufacturer guidance and was intended to standardize the perceived energy delivered to muscles of different masses and conditions.

Horses in the group s underwent a sham treatment consisting of the same preparation and the same protocol of the group t; however, the machine was not activated.

### 2.3. Pressure Algometry Evaluation

Pressure algometric measurements were conducted using a ZP series algometer (Baoshishan, Zhengzhou, China), equipped with a 2 mm flat metal tip calibrated to measure forces within a range of 0 to 50 N. All measurements were recorded in kilogram-force (kgf) units.

Pressure algometric measurements were performed the day before the first treatment (group t) or the sham procedure (groups s) to establish an initial baseline MNT (T0). Subsequently, measurements were carried out the day after each treatment session or sham procedure (T1 to T5), always between 11:00 am and 1:00 pm and before the horses were ridden. Additional measurements were then collected at 15-day intervals following the end of treatment or sham procedure, for a total of three further measurements of post-treatment (T6, T7, T8).

All measurements were conducted by the same operator, blinded to the treatment allocation, within the horses’ stable to minimize external distractions. The pressure algometer was firmly held against the skin with the left hand, while the right hand applied additional support to ensure stability and prevent slippage of the tip. Pressure was applied perpendicular to the skin surface at approximately a constant rate of 5 kg/cm^2^/s until a clear behavioral response to the pressure stimulus was elicited. This response included muscle fasciculation, cutaneous reflexes, active vertebral movement, or the horse moving away from the operator, as previously described [24,25,26]. Immediately after the first unequivocal response, pressure application was immediately stopped, and the corresponding value was recorded as the MNT. To improve reliability, measurements were repeated three consecutive times at each site. If one value differed markedly from the others, a fourth measurement was obtained, and the outlier was excluded. The mean of the three consistent measurements was used for statistical analysis.

**Figure 2:**
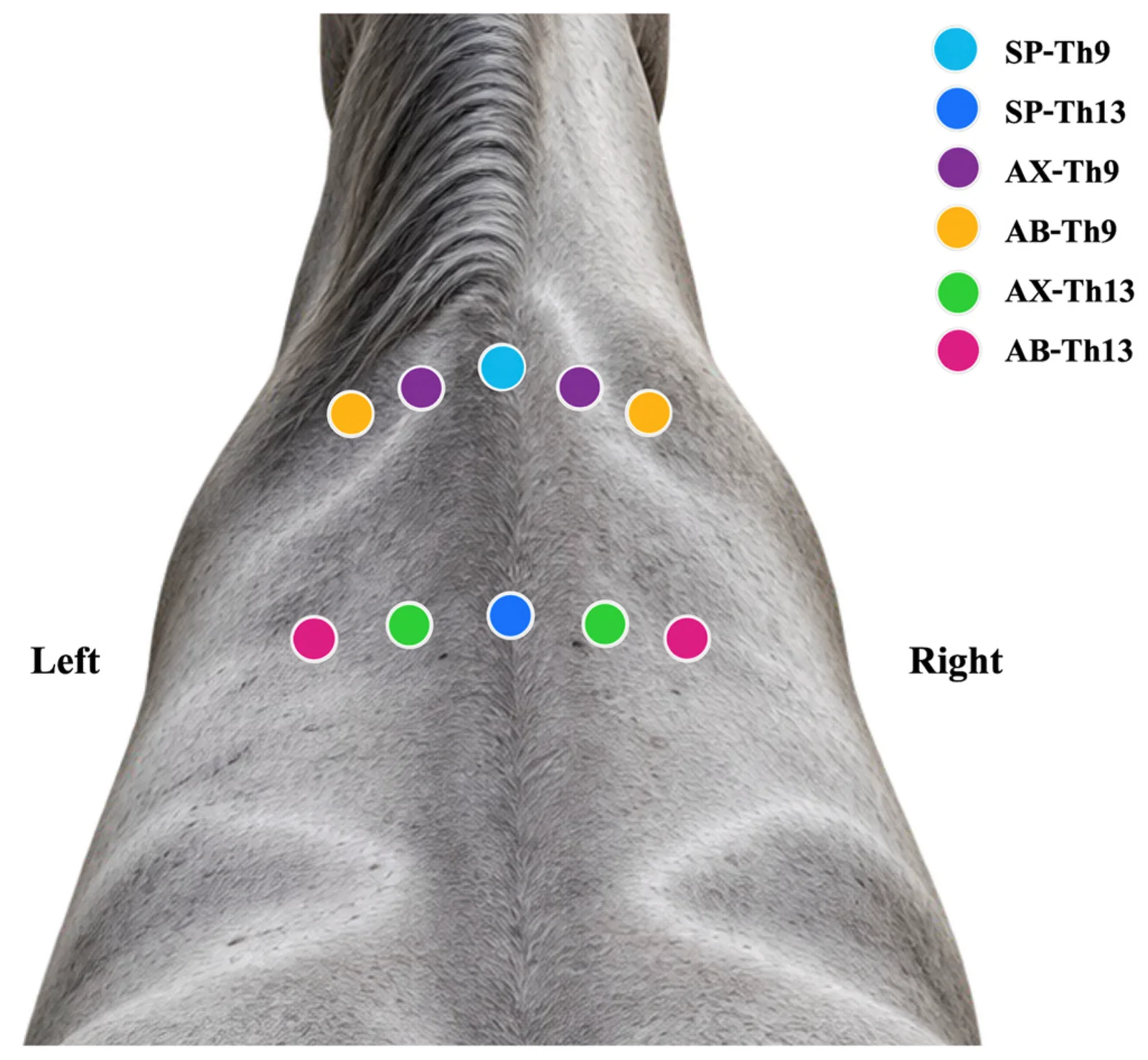
Schematic representation of the anatomical locations used for mechanical nociceptive threshold (MNT) measurements over the thoracolumbar region. Measurements were obtained at the level of the ninth (Th9) and thirteenth (Th13) thoracic vertebrae. At each level, five sites were assessed: one over the spinous process (SP), two axial sites (AX) located 4 cm lateral to the spinous process on the left and right sides, and two abaxial sites (AB) located 8 cm lateral to the spinous process on the left and right sides. Colors identify the corresponding measurement sites as indicated in the figure. Left and right refer to the horse’s anatomical sides.

Ten measurement sites were selected at predefined points along the back, bilaterally and symmetrically relative to the midline and axial skeleton of the spine. These sites were chosen based on anatomical landmarks described in previous studies and adapted for the purposes of the current study [25,27]. Each site was marked with an adhesive marker to ensure consistent measurements across repeated assessments. The MNTs were assessed in the area corresponding to the ninth (Th9) and thirteenth (Th13) thoracic vertebrae. For each region, ten MNT values were systematically examined, as shown in Table 1 and Figure 2.

### 2.4. Thermographic Images Acquisition

Thermographic measurements were obtained 20 min before and 5 min after each treatment or sham procedure using an FLIR One infrared thermography camera (Teledyne Flir, Limbiate, MI, Italy). Thermographic examinations were performed by the same operator according to the standardized protocol described in equine thermographic research [28,29,30]. Prior to image acquisition, the area of interest was carefully cleaned. All measurements were conducted in a controlled environment, avoiding direct sunlight and drafts to minimize artifacts from external environmental factors [31,32]. To avoid artifacts linked to skin compression or residual heating from electrode contact, images were acquired following methodological recommendations for equine thermography [28]. All examinations were conducted indoors to avoid environmental thermal fluctuations, and horses were allowed an acclimatization period of 15 min before imaging, as previously recommended [31]. In addition, to enhance reproducibility, all treatments were performed with identical device settings and standardized horse positioning [28].

**Figure 3:**
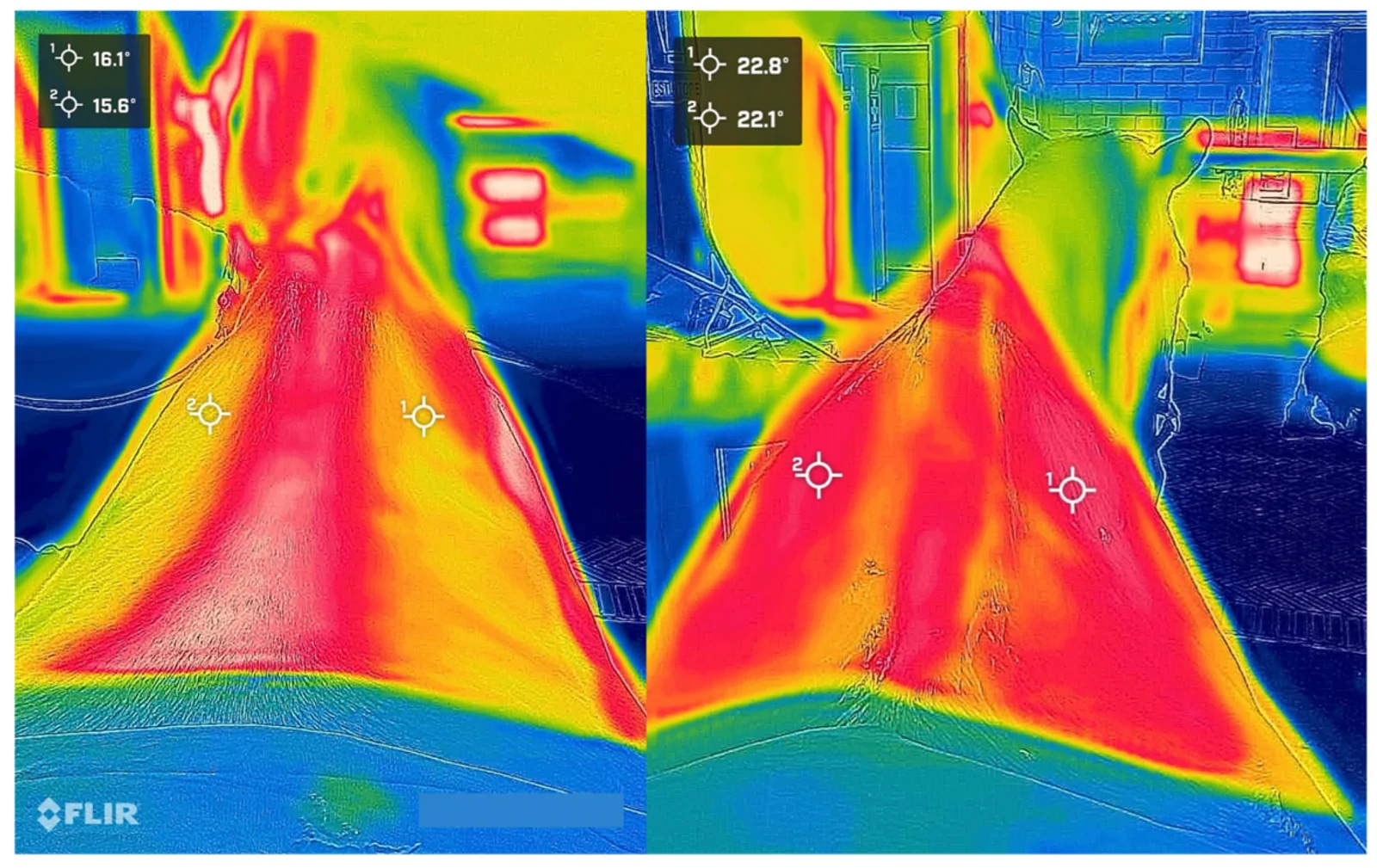
Thermographic images obtained before (on the left) and after (on the right) radiofrequency treatment in one horse included in the study.

Image acquisition was performed on the thoracolumbar region, approximately 90° relative to the horse, and from a distance of about two meters. Thermographic scans were conducted from an elevated, caudal position, maintaining a height of approximately 180 cm from the ground (Figure 3). Following image acquisition, thermal analysis was performed using the manufacturer’s software (Flir Tools^®^ Mobile, version 6.3.0, Limbiate, MI, Italy). Two identical measurement points (spot markers) were positioned bilaterally over the thoracolumbar epaxial musculature at the level of the tenth thoracic vertebra (Th10), approximately parallel to the dorsal midline (Figure 3). The mean value obtained from the two measurement points was used for statistical analysis.

The FLIR One camera has a thermal sensor resolution of 19,200 pixels and a thermal sensitivity of 70 mK, with a maximum temperature measurement capability of 400 °C. The camera is equipped with an internal mechanical shutter that periodically activates to perform calibration and optimize the image.

### 2.5. Statistical Analysis

All statistical analyses were performed in R version 4.5.3 (R Foundation for Statistical Computing, Vienna, Austria). Data analyses were performed using the dplyr (version 1.2.1) and tidyr (version 1.3.1) packages. Linear mixed-effects model was fitted using the nlme (version 3.1.168) package. Estimated marginal means and Holm-adjusted pairwise comparisons were obtained using the emmeans (version 2.0.4) package. Graphical visualizations were generated using ggplot2 (version 4.0.3), and model diagnostics were performed using the performance (version 0.15.2) package.

To minimize the influence of inter-individual baseline variability, absolute MNT values were converted to changes relative to baseline (ΔMNT). For each horse and anatomical site, ΔMNT was calculated as

ΔMNT = MNT_time_ − MNT_T0_

Thereby setting all baseline observations (T0) to zero.

The effect of treatment over time was evaluated using a linear mixed-effects model. Fixed effects included treatment group (group t vs. group s), time (T1–T8), anatomical site (SP-Th9, LAX-Th9, LAB-Th9, RAX-Th9 and RAB-Th9, SP-Th13, LAX-Th13, LAB-Th13, RAX-Th13 and RAB-Th13), and the interactions among treatment group, time, and anatomical site. Horse was included as a random effect to account for repeated observations within individuals, while a first-order autoregressive correlation structure was used to model temporal correlation among repeated measurements within each horse and anatomical site. Model assumptions were assessed by visual inspection of residual and normal Q–Q plots. Estimated marginal means were used for pairwise comparisons between treatment groups at each time point and between time points within each treatment group, with *p*-values adjusted using the Holm method.

For thermographic analysis, the change in skin surface temperature (ΔT) was calculated for each treatment session as the difference between post-treatment and pre-treatment temperatures (ΔT = POST − PRE). As each treatment session was considered an independent application, time was not included as a fixed effect. The effect of treatment on ΔT was evaluated using a linear mixed-effects model including treatment group as a fixed effect and horse as a random intercept to account for repeated measurements within individuals. Model assumptions were assessed by visual inspection of residual-versus-fitted and normal Q–Q plots, together with the Shapiro–Wilk test for residual normality and diagnostic assessment of residual variance using the performance package. Because the residual variance differed substantially between treatment groups, the final model was fitted using a heterogeneous residual variance structure (varIdent, nlme [version 3.1.168] package) to allow separate residual variances for each treatment group. Model adequacy was subsequently confirmed using normalized residuals. Estimated marginal means were calculated for each treatment group. Significance was set at *p* < 0.05.

## 3. Results

### 3.1. Horses

In the group t, there were three mares horses and seven geldings, with an age range from 8 to 21 years, breed included 8 warmbloods, one Anglo-Arabian and one pony. Five were competing at a high level (130–145 cm) and five at a low level (80–115 cm). Two horses had an MCS of 2, four horses an MCS of 3, and the remaining four an MCS of 4. No adverse effects were observed in any of the treated horses throughout the study.

In the group s, there are two mares and three geldings, with an age range from 9 to 18 years, breed included four warmblood and one pony. One was competing at high level, three at medium level (115–125 cm) and one at a low level. Two horses had an MCS of 2, two horses an MCS of 3 and the remaining one an MCS of 4.

### 3.2. Pressure Algometry

**Figure 4:**
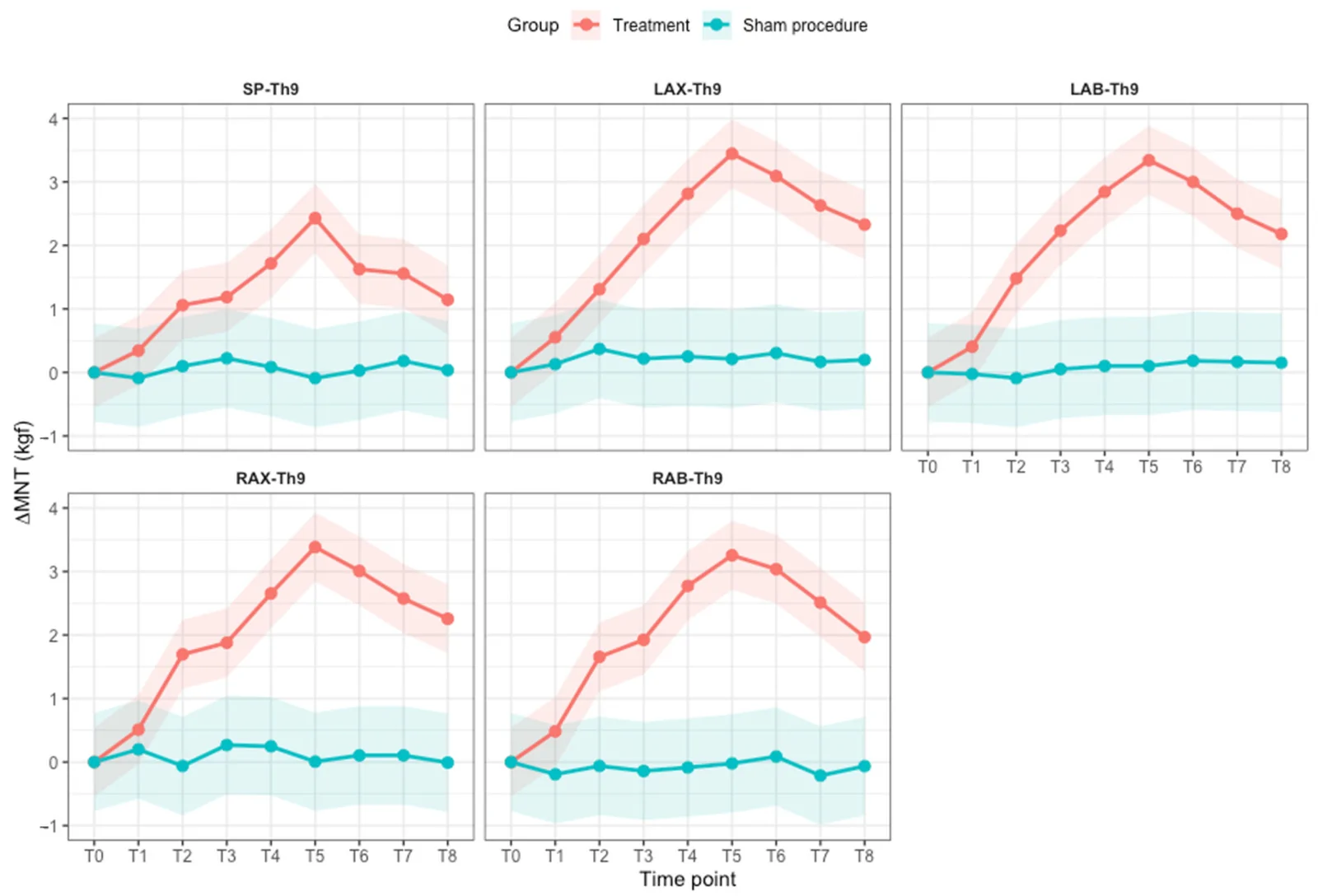
Estimated marginal means of changes in mechanical nociceptive threshold (ΔMNT) over time at the five anatomical sites evaluated at the Th9 level in the treatment (group t) and sham procedure (group s) groups. ΔMNT was calculated as the difference between each follow-up measurement and the corresponding baseline (T0) value for the same horse and anatomical site. Points indicate the estimated marginal mean at each time point, solid lines connect successive estimated marginal means, and shaded areas represent the corresponding 95% confidence intervals.

**Figure 5:**
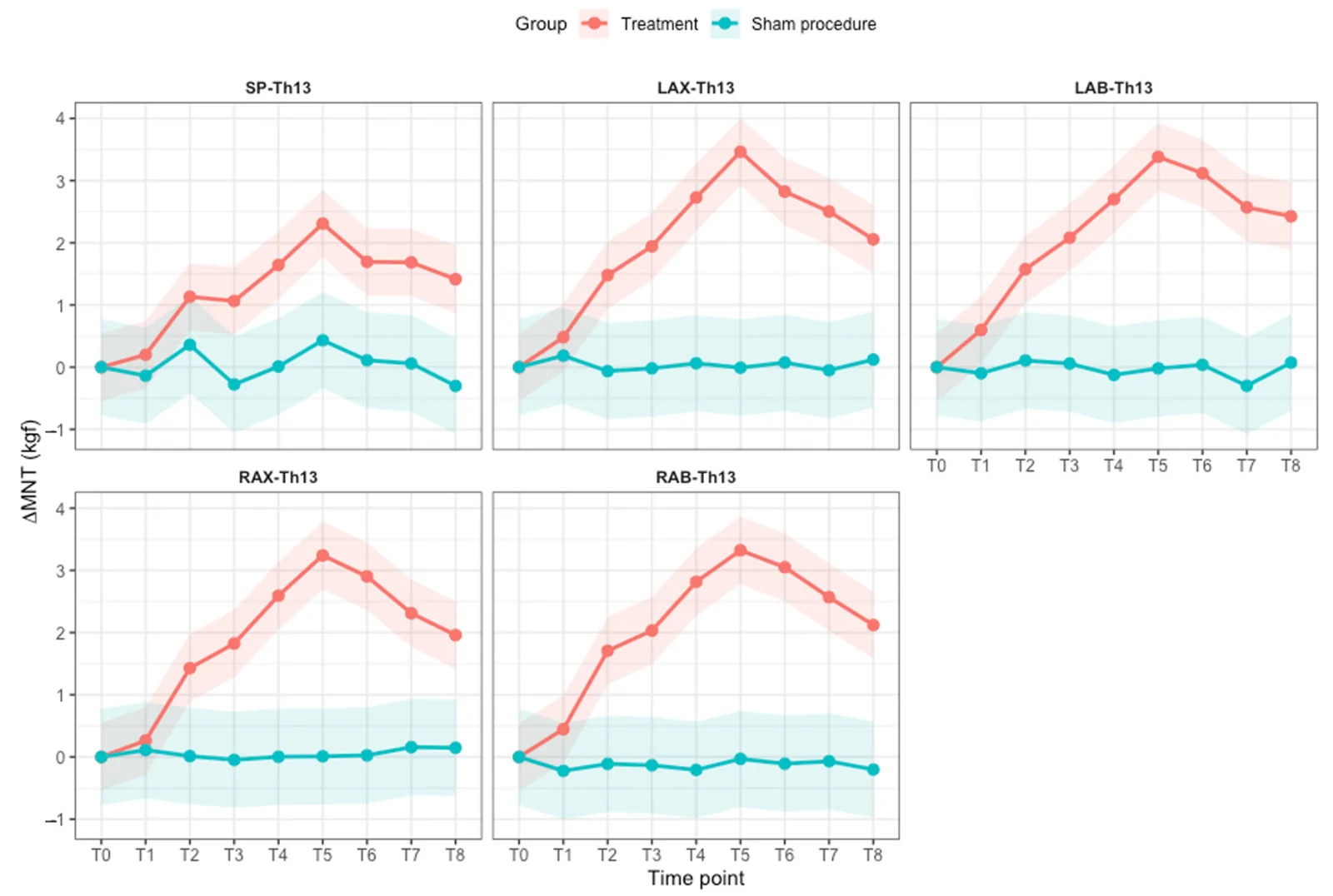
Estimated marginal means of changes in mechanical nociceptive threshold (ΔMNT) over time at the five anatomical sites evaluated at the Th13 level in the treatment (group t) and sham procedure (group s) groups. ΔMNT was calculated as the difference between each follow-up measurement and the corresponding baseline (T0) value for the same horse and anatomical site. Points indicate the estimated marginal mean at each time point, solid lines connect successive estimated marginal means, and shaded areas represent the corresponding 95% confidence intervals.

The details of the MNT obtained at the different sites at the level of Th9 and Th13 in group t and group s are summarized in Supplementary Tables S1 and S2; the temporal changes in ΔMNT at each anatomical site are shown in Figure 4 and Figure 5.

There was a significant effect of treatment group (*p* < 0.0001), time (*p* < 0.0001), and anatomical site (*p* = 0.0002), together with a significant treatment group × time interaction (*p* < 0.0001).

A small but significant treatment group × anatomical site interaction was also observed (*p* = 0.0465); however, neither the time × anatomical site interaction (*p* = 0.9077) nor the three-way interaction between treatment group, time and anatomical site (*p* = 0.9985) were significant, indicating that the temporal response to treatment was consistent across all anatomical sites (Figure 4 and Figure 5).

Following CRET treatment, horses in the group t showed a progressive increase in ΔMNT over time. The increase was evident after the first treatment session, reached its highest value around T5, and gradually declined thereafter, while remaining above baseline in the follow-up period. In contrast, horses in the group s showed minimal variation in ΔMNT throughout the study, with no significant temporal changes.

Pairwise comparisons demonstrated significantly greater ΔMNT values in the group t than in the group s from T2 onwards, whereas no significant difference was observed at T1 (Supplementary Table S3).

### 3.3. Thermographic Data

The mean (±SD) increase in skin temperature (ΔT) was 1.18 ± 3.19 °C in the group t and 0.78 ± 0.38 °C in the group s. No significant difference was detected between groups (*p* = 0.428). Estimated marginal means (95% CI) were 1.18 °C (0.19–2.16 °C) for the group t and 0.78 °C (0.41–1.14 °C) for group s.

## 4. Discussion

This study evaluated the effects of repeated monopolar capacitive CRET treatment on MNT in show-jumping horses with perceived chronic thoracolumbar pain. Consistent with the first hypothesis, five CRET sessions administered at 72 h intervals resulted in a progressive increase in MNT, with significantly greater ΔMNT values in treated horses compared with the sham group from the second treatment onwards. The effect peaked at T5, following competition of the treatment sessions, and subsequently showed a gradual decline during the follow-up period; however, MNT remained above pre-treatment values at the final assessment 45 days after treatment. This effect persisted throughout the 45-day follow-up period. The significant group × time interaction, together with the absence of a significant time × anatomical site or three-way interaction, indicates that the temporal response to treatment was consistent across the evaluated anatomical sites. Furthermore, the absence of comparable temporal changes in the sham group supports an association between the observed increase in MNT and CRET treatment rather than repeated testing, handling, or spontaneous temporal variation.

During the study period, exercise intensity was reduced in all horses, which could theoretically have influenced MNT independently of treatment. However, this potential confounding factor was controlled by including a sham group subjected to the same exercise restrictions, consisting of light flatwork, without active radiofrequency application. No significant changes in MNT were observed in the sham group over the course of the study, in contrast to the treated group. Therefore, the increase observed in treated horses is unlikely to be explained solely by the reduction in exercise intensity and is more likely associated with the therapeutic intervention.

CRET is based on the transfer of high-frequency alternating electrical currents to biological tissues and may induce both bioelectrical and thermal effects, depending on the physical characteristics of the applied signal and treatment parameters [8,9,10,11,12,13,14,15,16]. Experimental studies have described biological responses to capacitive–resistive radiofrequency exposure, including modulation of cellular activity and inflammatory processes, together with changes in local circulation and tissue metabolism [8,9,10,11,12,13,14,15,16]. Thermal effects associated with CRET have also been demonstrated and are influenced by treatment intensity, electrode configuration, tissue electrical properties, and application technique [9,23]. These mechanisms may contribute to changes in tissue function and nociceptive sensitivity [9,10,11,12,13,14,15,16,23]; however, the present study was not designed to investigate the biological mechanisms underlying the observed increase in MNT.

The temporal pattern observed in the treatment group is particularly noteworthy. ΔMNT progressively increased during the treatment period, reached its highest values around the final treatment session, and subsequently declined while remaining above baseline throughout the follow-up period. This pattern suggests that repeated CRET applications may have a cumulative effect on mechanical nociceptive sensitivity rather than producing only a short-lived response to each individual session. The absence of a comparable pattern in the sham group further supports this interpretation. Nevertheless, the mechanisms responsible for the persistence of the effect cannot be established from the present study and require further investigation.

Previous studies using capacitive–resistive radiofrequency in horses have reported heterogeneous effects on pain and musculoskeletal function [13,17,18,19,20,21,33]. In horses with cervical pain and dysfunction, six treatments administered twice weekly over three weeks did not significantly modify MNT, visual analog scores, cervical pain and stiffness, range of motion, or forelimb function [33]. This contrasts with the progressive MNT response observed in the present study. However, the previous protocol combined capacitive and resistive applications in continuous mode, whereas the present study employed a monopolar capacitive pulsed protocol. Differences in the anatomical region treated, study populations, treatment mode, electrode configuration, intensity, duration, and interval between applications may therefore contribute to the apparently contrasting findings [33]. These discrepancies suggest that clinical responses to radiofrequency-based therapies may be highly dependent on the specific treatment parameters employed and reinforce the need to define optimal dosimetry and treatment schedules in horses.

Conversely, previous findings in horses with thoracolumbar pain provide clinical support for a beneficial effect of repeated CRET treatment on back sensitivity [18]. Four sessions of 448 kHz CRET administered over two consecutive weeks resulted in a significant reduction in thoracolumbar pain assessed by palpation, while no significant change was detected following the sham procedure [18]. Epaxial muscle pain also decreased after active treatment, although a smaller improvement was observed in the sham group [18]. The same study reported increases in several accelerometric variables, particularly dorsoventral power, which were interpreted as suggestive of improved back flexibility [18]. However, dorsoventral displacement did not significantly change following either active or sham treatment, and the biomechanical evidence for increased spinal flexibility was therefore indirect [18]. Although direct comparison is limited by the use of a different device and a combined capacitive–resistive protocol at 448 kHz, these clinical findings are consistent with the reduced mechanical sensitivity observed in the present study. The current results extend these observations by providing a quantitative assessment based on pressure-derived MNT and by demonstrating an effect that peaked at T5 and subsequently declined, while remaining above the baseline throughout a 45-day follow-up period.

A predominantly immediate response has also been described following conventional TECAR treatment of the thoracolumbar musculature [20]. A single capacitive–resistive treatment produced a marked reduction in palpation-assessed *longissimus dorsi* muscle tone that was evident immediately after treatment and maintained 10 min later [20]. Although palpation scores and MNT assess different aspects of the response to treatment and cannot be directly compared, both outcomes provide evidence of changes in thoracolumbar muscle sensitivity or response following radiofrequency-based interventions. Interestingly, this immediate clinical response was accompanied by a substantial increase in skin surface temperature, whereas the progressive and sustained increase in MNT observed in the present study occurred without a treatment-specific superficial thermal response. Together, these findings suggest that different radiofrequency delivery protocols may produce distinct temporal and thermal patterns of response and that a marked increase in superficial temperature may not be required for the effects observed following repeated pulsed capacitive treatment.

Pressure algometry was used to provide an objective assessment of mechanical nociceptive sensitivity. The decision not to include immediate pre- and post-treatment measurements was made to minimize handling time and avoid repeated nociceptive stimulation of the same anatomical region, which may itself modulate the threshold through sensitization or habituation [25,26,34,35]. Pressure algometry is a reliable and non-invasive method for assessing pain sensitivity and mechanical nociception in horses [25,26,34,35]. Although pressure algometry results can vary across individuals and settings, the technique is particularly useful for intra-individual comparisons, which represented the primary focus of the present study [34]. Conversion of absolute MNT values to changes from each horse’s baseline further reduced the influence of inter-individual variability. Moreover, the stability of MNT in the sham group reduces the likelihood that the progressive increase observed in treated horses resulted from habituation to repeated algometric testing.

Thermographic assessment showed an increase in skin surface temperature after both active CRET and sham procedures; however, the magnitude of temperature change did not differ significantly between groups. Therefore, the immediate superficial thermal response observed in the present study cannot be specifically attributed to radiofrequency energy delivery. Factors common to both procedures, including repeated movement of the applicator over the skin, electrode and conductive-gel contact, and local mechanical stimulation, may have contributed to this response.

The thermal response reported following radiofrequency-based therapies in horses appears to vary substantially according to the treatment protocol [19,20]. A marked increase in surface temperature has been demonstrated after TECAR treatment of the *longissimus dorsi*, with temperatures significantly higher than those in sham-treated horses both immediately after treatment (3.1 °C) and 10 min later [20]. That protocol combined capacitive and resistive modes at 500 kHz and was intended to induce endogenous tissue heating [20]. Conversely, thermographic evaluation of different CRET intensities showed no significant differences in mean or maximum skin temperature between sham and low-intensity treatment, whereas moderate- and high-intensity protocols produced substantially greater thermal responses; some thermal changes remained detectable up to 30 min after treatment [19]. The similarity between sham and low-intensity applications also suggests that part of the small superficial temperature increase observed under these conditions may derive from mechanical factors associated with the application itself rather than from radiofrequency energy alone [19].

Taken together, these findings indicate that superficial heating during CRET is strongly dependent on treatment parameters and should not be generalized across devices or protocols. Frequency, intensity and energy delivery, capacitive and/or resistive application, treatment duration, electrode configuration, application technique, and device-specific characteristics may all influence the resulting thermal response. Direct quantitative comparisons with the present protocol should therefore be made cautiously. Importantly, the progressive and sustained increase in MNT observed in the present study occurred despite the absence of a treatment-specific increase in skin surface temperature, suggesting that a marked superficial thermal response may not be necessary for the observed effect of repeated pulsed capacitive CRET. This finding should not, however, be interpreted as evidence of an exclusively non-thermal mechanism. Infrared thermography measures skin surface temperature and cannot directly assess temperature changes within the underlying epaxial musculature; consequently, deeper thermal effects cannot be excluded [9,23].

It should also be acknowledged that ambient temperature and the potential cooling effect of the conductive gel were not directly measured and may have influenced thermographic readings. However, assessments were conducted under controlled indoor conditions following a standardized acclimatization period and with consistent measurement timing to minimize environmental variability. The conductive gel was maintained at ambient temperature, and the same preparation and application procedures were used in both groups.

A customized CRET protocol was employed in this study, using a constant frequency of 500 kHz with power levels of 30–35% adjusted according to individual muscle condition. This approach is conceptually similar to applications in human medicine, where treatment parameters may be adjusted according to the electrical impedance of the treated tissues to optimize energy delivery and minimize excessive thermal exposure [15]. In human physiotherapy, parameter adjustment is also commonly complemented by the patient’s subjective perception of heat, which allows the operator to modulate treatment intensity according to individual tolerance. Although this feedback does not represent a standardized dosimetry measure, it provides additional information for safe application [9,23]. Such subjective feedback is unavailable in equine patients. Consequently, the protocol used in the present study relied on predefined device settings adjusted according to muscle condition, together with strict control of operator-dependent variables, including electrode pressure, movement speed, and contact preparation, to promote consistent energy delivery [28,31]. Further studies are required to determine optimal treatment intensity, frequency of application, and overall treatment schedules for CRET therapy in horses.

Several limitations should be considered. Horses were included on the basis of history of chronic back pain supported by clinical examination; however, clinical assessment of pain by manual palpation inherently includes a degree of subjectivity. Moreover comprehensive diagnostic investigation was not performed to identify a specific underlying thoracolumbar pathology. Saddle fit and horse–rider interaction were also not specifically evaluated and may have contributed to the clinical presentation. Therefore, the study population should be interpreted as horses with perceived chronic thoracolumbar pain rather than horses affected by a specific diagnosed back disorder. In addition, the relatively small study population was heterogeneous in terms of age, breed, competition level, and muscle condition score. This heterogeneity may have introduced additional variability and potentially influenced the observed outcomes, and should therefore be considered when interpreting the results of the study.

Another limitation concerns the timing of MNT measurements. A baseline measurement was obtained before the beginning of the treatment cycle, whereas subsequent measurements were performed the day after each treatment or sham session rather than immediately before and after each individual application. Consequently, the study design does not allow determination of the magnitude of the immediate response to each treatment or whether MNT partially returned towards baseline between consecutive sessions. A pre- and post-session design would provide a more detailed characterization of these short-term temporal dynamics and should be considered in future studies.

Nevertheless, based on the overall pattern observed in the treatment group, this limitation is unlikely to substantially affect the main interpretation of the results. While a pre- and post-session design would have strengthened characterization of the short-term response, the current protocol supports the conclusion that repeated CRET treatments were associated with a progressive and sustained increase in MNT.

Finally, MNT was the single outcome used to assess the response to treatment. Although it provided a quantitative measure of mechanical nociceptive sensitivity, additional functional outcomes, such as muscle tone or thoracolumbar flexibility, were not objectively assessed. Therefore, potential treatment effects on other aspects of thoracolumbar function may have been missed and should be investigated in future studies.

## 5. Conclusions

In conclusion, five monopolar capacitive CRET treatments administered at 72 h intervals were associated with increased mechanical nociceptive thresholds in show-jumping horses with perceived chronic thoracolumbar pain, with differences from the sham group persisting throughout the 45-day follow-up period. Skin surface temperature increased after both active and sham procedures, with no significant difference between groups, indicating that the immediate superficial thermal response could not be specifically attributed to the pulsed monopolar capacitive radiofrequency energy delivery. Further studies are warranted to determine the mechanisms underlying the observed MNT response and to establish optimal treatment parameters and schedules in horses.

## Figures and Tables

**Table 1:** Details of the sites where mechanical nociceptive thresholds were tested along the back, positioned. Except for those located at the spinous processes, the sites were bilaterally and symmetrically on each side of the thoracolumbar region.

Measurement Site	Anatomical Area
SP-Th9	Spinous process of Th9
LAX-Th9	Left axial area at the level of Th9, 4 cm laterally to SP
RAX-Th9	Right axial area at the level of Th9, 4 cm laterally to SP
LAB-Th9	Left abaxial area at the level of Th9, located 8 cm laterally to SP
RAB-Th9	Right abaxial area at the level of Th9, located 8 cm laterally to SP
SP-Th13	Spinous process of Th13
LAX-Th13	Left axial area at the level of Th13, 4 cm laterally to SP
RAX-Th13	Right axial area at the level of Th13, 4 cm laterally to SP
LAB-Th13	Left abaxial area at the level of Th13, located 8 cm laterally to SP
RAB-Th13	Right abaxial area at the level of Th13, located 8 cm laterally to SP

SP = spinous process; Th9 = ninth thoracic vertebra; Th13 = thirteen thoracic vertebra.

## Data Availability

The raw data supporting the conclusions of this article will be made available by the authors on request.

## Supplementary Materials

The following supporting information can be downloaded at https://www.mdpi.com/article/10.3390/ani16182943/s1, Table S1: Mechanical nociceptive threshold values obtained at the different time point, from T0 to T8, at the level of ninth thoracic vertebra (Th9), expressed as mean ± standard deviation or median and range, as appropriate in the treatment group (group t) and in the sham procedure group (group s); Table S2: Mechanical nociceptive threshold values obtained at the different time point, from T0 to T8, at the level of thirteen (Th13) thoracic vertebra, expressed as mean ± standard deviation or median; range, as appropriate in the treatment group (group t) and in the sham procedure group (group s); Table S3: Pairwise comparisons of the estimated marginal mean differences in changes in mechanical nociceptive threshold (ΔMNT) between the treatment and sham procedure groups at each time point. Estimated mean differences are expressed as treatment minus sham procedure and are averaged across all anatomical sites. Positive values indicate a greater increase in ΔMNT in the treatment group. *p*-values were adjusted using the Holm method.

## Abbreviations

The following abbreviations are used in this manuscript:

CRET	Capacitive Resistive Electrical Transfer
CRMRF	Capacitive Resistive Monopolar Radiofrequency
MCS	Muscle Condition Score
MNT	Mechanical Nociceptive Threshold
