# Supplementary material for: Effects of Repeated Monopolar Pulsed Capacitive Radiofrequency Energy Treatments on Mechanical Nociceptive Thresholds and Skin Temperature in Show-Jumping Horses with Thoracolumbar Pain
Source: Animals (Basel). 2026 Sep 19;16(18):2943. doi: 10.3390/ani16182943 (PMC13603132; doi:10.3390/ani16182943)
Supplement: Supplementary file 1 [file animals-16-02943-s001.zip › animals-4547776-supplementary.pdf]

Supplementary Table S1. Mechanical nociceptive threshold values obtained at the different time point, from T0 to T8, at the level of ninth thoracic vertebra (Th9), expressed as mean ± standard deviation or median and range, as appropriate in the treatment group (*group t*) and in the sham procedure group (*group s*).

	**Location**				
	**SP-Th9**	**LAX-Th9**	**LAB-Th9**	**RAX-Th9**	**RAB-Th9**
**Time**	***Group t***				
**T0**	1.997 ± 1.003	1.357 ± 0.586	1.525 ± 0.564	1.359 ± 0.582	1.577 ± 0.422
**T1**	2.341 ± 0.828	1.911 ± 0.676	1.929 ± 0.772	1.756; 1.331 – 3.566	1.675; 1.292 – 3.450
**T2**	3.057 ± 1.261	2.667 ± 1.020	3.006 ± 1.127	3.054 ± 1.258	3.005 ± 1.038
**T3**	3.099; 2.057 – 4.468	3.462; 1.875 – 5.022	3.759 ± 1.265	3.237 ± 1.095	3.400 ± 0.960
**T4**	3.712 ± 0.931	4.172 ± 0.954	4.368 ± 0.936	4.012 ± 0.841	4.170 ± 0.881
**T5**	4.472 ± 1.016	5.172; 2.338 – 5.390	5.166; 2.697 – 5.509	5.001; 2.440 – 5.374	5.225; 2.490 – 5.400
**T6**	3.623 ± 1.082	4.551; 2.224 – 5.242	4.745; 2.150 – 5.239	4.514; 2.617 – 4.869	4.682; 2.362 – 5.169
**T7**	3.553 ± 1.198	4.337; 0.985 – 5.174	4.303; 1.231 – 5.239	4.125; 1.233 – 5.026	4.204; 0.853 – 4.892
**T8**	3.141 ± 1.261	3.992; 0.867 – 4.597	3.997; 1.212 – 4.817	3.945; 0.987 – 4.865	3.934; 0.865 – 4.296
	Group s				
**Time**					
**T0**	2.887 ± 1.341	2.906 ± 1.130	3.063 ± 1.05	3.038 ± 1.071	3.039 ± 1.239
**T1**	2.798 ± 1.24	3.037 ± 1.177	3.038 ± 1.312	3.238 ± 1.186	2.846 ± 1.251
**T2**	2.986 ± 1.457	3.277 ± 1.215	2.973 ± 1.323	2.977 ± 0.931	2.978 ± 1.236
**T3**	3.109 ± 1.408	3.123 ± 1.524	3.115 ± 1.256	3.306 ± 1.066	2.898 ± 1.328
**T4**	2.971 ± 1.355	3.157 ± 1.519	3.164 ± 1.254	3.286 ± 1.178	2.954 ± 1.253
**T5**	2.796 ± 1.417	3.118 ± 1.4	3.163 ± 1.134	3.044 ± 1.094	3.017 ± 1.009
**T6**	2.916 ± 1.417	3.212 ± 1.296	3.246 ± 1.047	3.144 ± 1.191	3.126 ± 1.158
**T7**	3.067 ± 1.346	3.073 ± 1.342	3.23 ± 1.247	3.144 ± 1.09	2.826 ± 1.25
**T8**	2.923 ± 1.340	3.104 ± 1.217	3.215 ± 1.13	3.029 ± 1.191	2.975 ± 1.388

Supplementary Table S2. Mechanical nociceptive threshold values obtained at the different time point, from T0 to T8, at the level of thirteen (Th13) thoracic vertebra, expressed as mean ± standard deviation or median; range, as appropriate in the treatment group (*group t*) and in the sham procedure group (*group s*).

	**Location**				
	**SP-Th13**	**LAX-Th13**	**LAB-Th13**	**RAX-Th13**	**RAB-Th13**
**Time**	***Group t***				
**T0**	1.927; 1.012 – 4.591	2.214 ± 0.485	1.446 ± 0.367	1.577 ± 0.422	1.466 ± 0.367
**T1**	2.415 ± 0.581	1.886 ± 0.673	2.064 ± 0.848	1.675; 1.292 – 3.450	2.064 ± 0.848
**T2**	3.347 ± 1.090	2.884 ± 1.067	3.041 ± 1.126	3.005 ± 1.038	3.041 ± 1.126
**T3**	3.279 ± 0.826	3.348 ± 1.260	3.548 ± 1.232	3.400 ± 0.960	3.548 ± 1.232
**T4**	3.857 ± 0.837	4.132 ± 0.872	4.167 ± 0.894	4.170 ± 0.881	4.167 ± 0.894
**T5**	4.716; 2.434 – 5.340	5.129; 2.482 – 5.797	5.117; 2.637 – 5.539	5.225; 2.490 – 5.400	5.117; 2.637 – 5.539
**T6**	3.909 ± 0.878	4.459; 2.352 – 4.922	4.745; 2.578 – 5.376	4.682; 2.362 – 5.169	4.745; 2.578 – 5.376
**T7**	4.087; 1.359 – 4.905	4.168; 1.043 – 4.949	4.256; 0.878 – 5.118	4.204; 0.853 – 4.892	4.256; 0.878 – 5.118
**T8**	3.936; 1.203 – 4.769	3.779; 1.006 – 4.275	4.006; 0.956 – 4.893	3.934; 0.865 – 4.296	4.006; 0.956 – 4.893
	Group s				
**Time**					
**T0**	3.337 ± 1.356	2.982 ±1.058	3.062 ± 1.187	2.971 ± 1.078	3.679; 1.888 – 3.956
**T1**	3.2 ± 1.162	3.167 ± 1.023	2.964 ± 1.096	3.891; 1.695 – 4.113	2.866 ± 1.171
**T2**	3.697 ± 1.081	2.919 ± 0.978	3.17 ± 1.082	2.985 ± 0.914	2.979 ± 1.301
**T3**	3.06 ± 1.046	2.963 ± 1.065	3.121 ± 0.991	3.728; 1.462 – 3.768	2.956 ± 1.373
**T4**	3.347 ± 1.094	3.045 ± 1.391	2.938 ± 1.323	2.974 2.997 ± 1.226	3.689; 1.328 – 3.76
**T5**	3.769 ± 1.232	2.974 ± 0.915	3.043 ± 1.159	3.819; 1.389 – 4.006	3.058 ± 1.245
**T6**	3.448 ± 1.077	3.057 ± 1.271	3.101 ± 0.989	2.997 ± 0.905	2.981 ± 1.097
**T7**	3.397 ± 0.907	2.932 ± 1.244	2.763 ± 1.628	3.129 ± 1.29	3.021 ± 1.185
**T8**	3.036 ± 0.892	3.104 ± 0.928	3.135 ± 1.017	3.119 ± 1.075	2.887 ± 1.182

Supplementary Table S3. Pairwise comparisons of the estimated marginal mean differences in changes in mechanical nociceptive threshold (ΔMNT) between the treatment and sham procedure groups at each time point. Estimated mean differences are expressed as treatment minus sham procedure and are averaged across all anatomical sites. Positive values indicate a greater increase in ΔMNT in the treatment group. P-values were adjusted using the Holm method.

Time point	Estimated mean difference (kgf)	SE	95% CI	Holm-adjusted P-value
**T0**	na	na	na	na
**T1**	0.442	0.266	-0.133 to 1.018	0.121
**T2**	1.396	0.266	0.821 to 1.971	0.0002
**T3**	1.807	0.266	1.231 to 2.382	<0.0001
**T4**	2.494	0.266	1.918 to 3.069	<0.0001
**T5**	3.098	0.266	2.523 to 3.673	<0.0001
**T6**	2.650	0.266	2.075 to 3.225	<0.0001
**T7**	2.320	0.266	1.745 to 2.896	<0.0001
**T8**	1.970	0.266	1.395 to 2.545	<0.0001
SE, standard error; CI, confidence interval.				
